# Research progress of nano delivery systems for intraocular pressure lowering drugs

**DOI:** 10.1016/j.heliyon.2024.e32602

**Published:** 2024-06-12

**Authors:** Xiaoyu Zhou, Dengming Zhou, Xinyue Zhang, Yang Zhao, Li Liao, Ping Wu, Baihua Chen, Xuanchu Duan

**Affiliations:** a Aier Glaucoma Institute, Hunan Engineering Research Center for Glaucoma with Artificial Intelligence in Diagnosis and Application of New Materials, Changsha Aier Eye Hospital, Changsha, Hunan, China; b Eye Hospital, Wenzhou Medical University, Wenzhou, Zhejiang, China; c The Second Xiangya Hospital, Central South University, Changsha, Hunan Province, China

**Keywords:** Glaucoma, Intraocular pressure lowering drugs, Nanotechnology, Medication delivery systems

## Abstract

Glaucoma is a chronic ocular disease characterized by optic atrophy and visual field defect. The main risk factor for glaucoma onset and progression is elevated intraocular pressure, which is caused by increased aqueous humor outflow resistance. Currently, the primary method for glaucoma therapy is the use of intraocular pressure lowering drugs. However, these drugs, when administered through eye drops, have low bioavailability, require frequent administration, and often result in adverse effects. To overcome these challenges, the application of nanotechnology for drug delivery has emerged as a promising approach. Nanoparticles can physically adsorb, encapsulate, or chemically graft drugs, thereby improving their efficacy, retention time, and reducing adverse reactions. Moreover, nanotechnology has opened up new avenues for ocular administration. This article provides a comprehensive review of nano systems for intraocular pressure lowering drugs, encompassing cholinergic agonists, β-adrenergic antagonists, α-adrenergic agonists, prostaglandin analogs, carbonic anhydrase inhibitors, Rho kinase inhibitors, and complex preparations. The aim is to offer novel insights for the development of nanotechnology in the field of intraocular pressure lowering drugs.

## Graphical abstract

**Figure:**
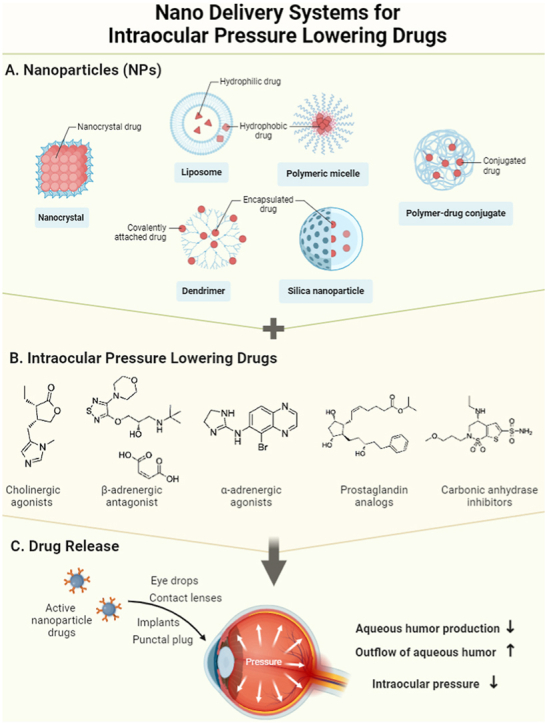

## 1 Introduction

Glaucoma is a major cause of irreversible blindness globally. However, its treatment still encounters significant challenges and demands sustained efforts. The most effective clinical approach to managing glaucoma is by controlling intraocular pressure, which can be accomplished through medications, laser treatment, and surgery [1]. The prolonged use of topical intraocular pressure lowering drugs can result in various ocular adverse reactions, including dry eye syndrome, chronic allergic conjunctivitis, and meibomian gland dysfunction. These adverse reactions primarily occur due to the limited effectiveness of eye drops and the inclusion of preservatives, which can lead to reduced compliance and increased frequency of drug administration [2]. Benzalkonium chloride (BAK) is the most commonly used preservative in eye drops, but it can have negative effects on the cornea and conjunctiva, leading to ocular surface disease [3]. To address this issue, there are some preservative (including BAK)-free preparations of topical ocular drugs for treating glaucoma available, such as Tapros and IYUZEH. Researchers are also working on developing composite formulations, novel ophthalmic formulations, and innovative drug storage devices to minimize the adverse reactions of anti-glaucoma medications. In recent years, nanomaterials have gained significant attention as a rapidly advancing class of materials. In order to reduce ocular surface disease caused by topical intraocular pressure lowering drugs, several researchers have focused on developing pharmaceutical nanoparticles [4]. Nanoparticles are materials typically defined as being between 1 and 1000 nm in size, and possess unique structural, chemical, mechanical, magnetic, and biological properties.

Chronic ocular diseases, such as glaucoma, diabetic retinopathy, and uveitis, often necessitate long-term medication therapy. However, natural compound drugs face several challenges, including inadequate stability in the body, low bioavailability, poor water solubility, limited absorption, and lack of targeted delivery [5]. In order to overcome these limitations, researchers have developed a method of encapsulating and/or adsorbing drugs onto nanoparticles. This approach significantly improves the water solubility and bioavailability of drugs, while also enabling targeted delivery and controlled release [4]. Furthermore, certain nanoparticles possess an imaging function that enables researchers to track the real-time release, distribution, and absorption of drugs. The initial generation of nanomedicine delivery systems consisted of liposomes or micelles containing anti-tumor drugs, which have already received FDA approval and are being used in clinical applications. For instance, the earliest liposome formulation of doxorubicin (DOXIL) comprises liposomes that encapsulate the natural compound doxorubicin, with a phospholipid molecular layer on the surface modified with polyethylene glycol (PEG) [6]. The outer nanostructures of DOXIL enable it to evade recognition and phagocytosis by immune cells such as monocytes and macrophages. This mechanism prolongs the drug's circulation time in the bloodstream. The tumors' enhanced permeability and retention (EPR) effect allows DOXIL to accumulate in cancerous tissue for an extended period, thereby enhancing its efficacy in killing carcinoma cells. Moreover, the phospholipid molecular shell structure of DOXIL reduces its affinity for cardiac tissue, minimizing its toxic and adverse effects on the heart.

Nano delivery systems have been extensively researched and developed in the field of ophthalmology to lower intraocular pressure [7]. One common nano delivery system used in ophthalmology is the nanoparticle carrier, such as nanospheres, nanoparticles, or nanomicelles [8]. These carriers serve to encapsulate drugs that lower intraocular pressure, thereby improving their solubility, stability, and permeability. Additionally, they can prolong the presence of drugs in ocular tissues and decrease their elimination rate. By controlling factors like size, surface properties, and drug release mechanisms, sustained and controlled drug release can be achieved, ultimately enhancing the therapeutic effects of the drugs. Moreover, nano delivery systems can precisely target specific locations within the eye, such as the ciliary body or the anterior chamber angle, leading to improved local efficacy, reduced side effects, and minimized systemic drug absorption [9,10]. Furthermore, novel nanomaterials like carbon nanotubes, graphene, and metal nanoparticles have been utilized in nano delivery systems for intraocular pressure-lowering drugs due to their unique physical and chemical properties that enhance drug penetration and absorption, providing a more effective platform for drug delivery [11,12]. Some ongoing studies are also focused on developing multi-drug delivery systems that combine different types of glaucoma medications, aiming to achieve the benefits of combination therapy [13]. This integrated approach can improve efficacy, reduce side effects, and simplify medication for patients.

The applications of nanotechnology in anti-glaucoma therapy extend to preventing scarring after filtration surgery. Hu et al. used amniotic membrane loaded with 5-fluorouracil (5-Fu) poly (lactic-*co*-glycolic acid) (PLGA) nanoparticles to inhibit the formation of scarring in filtering blebs [14]. Jiang et al. designed a cationic dendrimer poly (amidoamine) (PAMAM) nanoparticle to deliver complexes of 5-Fu and anti-TGF-β oligonucleotide, aiming to prevent fibrosis after trabeculectomy [15]. Additionally, a PVA slow-release membrane loaded with 5-Fu graphite oxide and silver nanoparticles was developed to regulate the anti-scarring ability of the filtering bleb [16]. In our previous study, we also developed a novel adjustable PHBV basement film to enhance the effectiveness of glaucoma surgery by inhibiting scar formation [17].

**Fig. 1:**
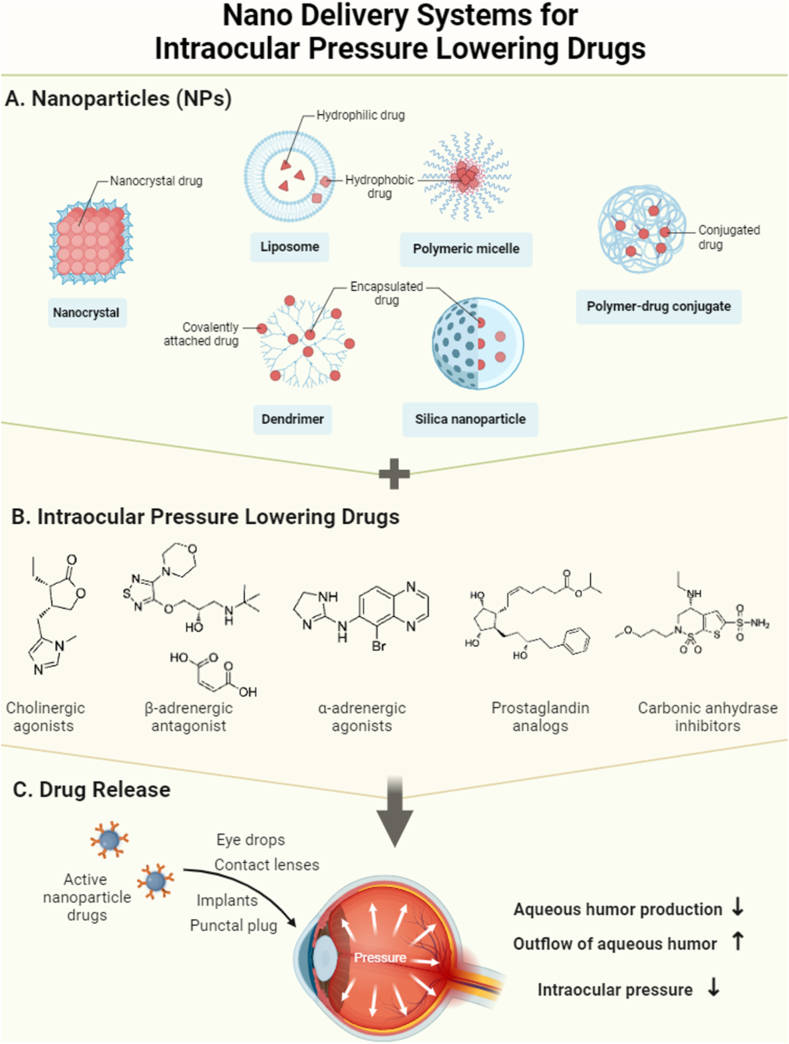
Nanomedicine delivery systems of various IOP lowering drug types.

The classification of topical intraocular pressure lowering medications typically includes cholinergic agonists, β-adrenergic antagonists, α-adrenergic agonists, prostaglandin analogs (PGAs), carbonic anhydrase inhibitors (CAIs), Rho-associated coiled-coil forming protein kinase (ROCK) inhibitors, and composite preparations. The field of ophthalmology is actively researching and applying nano delivery systems for anti-glaucoma medications. These systems show promise in improving long-term medication compliance, reducing adverse effects, and increasing the success rate of surgical treatment. This article aims to review the current research progress of nanomedicine delivery systems based on different drug types (Table 1, Fig. 1).

**Table 1:** Case studies of nano delivery systems for intraocular pressure lowering drugs.

author	year	drugs	carriers	Dosage forms	advantages	Stage (Clinical/Animal/In vitro)
Diebold et al.	1989	pilocarpine	PBCA(polybutyl-2-cyanoacrylate) NPs	drops	Extend drug action time (sustained-release)	clinical
Kao et al.	2006	pilocarpine	chitosan carbopol NPs	drops	Extend drug action time (sustained-release)	*in vitro*
Elsa et al.	2018	pilocarpine	polymer nanomicelles NPs	drops	pH-Dependent Release	*in vitro*
Lin et al.	2006	pilocarpine	polyacrylic acid NPs	drops	Extend drug action time (sustained-release)	animal
Nguyen et al.	2020	pilocarpine	PLA (polylactic acid) NPs	drops	Extend drug action time (sustained-release)	animal
Luo et al.	2020	pilocarpine	Ce-CS(Cerium dioxide with surface-modified chitosan) NPs	drops	Enhance corneal penetration ability, Targeted drug release	animal
Nguyen et al.	2022	pilocarpine	Ce-CS NPs	drops	Enhance corneal penetration ability, Targeted drug release	animal
Agnihotri et al.	2007	Timolol	chitosan NPs	drops	Extend drug action time (sustained-release)	*in vitro*
Attama et al.	2009	Timolol	solid lipid NPs	drops	Enhance corneal penetration ability	*in vitro*
Hyun et al.	2012	Timolol	PGT (propoxylated glyceryl triacylate) NPs	contact lenses	Extend drug action time (sustained-release)	*in vitro*
Hyun et al.	2013	Timolol	*p*-HEMA (poly hydroxyl ethyl methacrylate) NPs	Fornix Insert	Extend drug action time (sustained-release)	*in vitro*
Gallarate et al.	2012	Timolol	Aerosol oT (AOT) nanoemulsion	drops	Enhance corneal penetration ability	*in vitro*
Vijaya Rani et al.	2022	Timolol	Carbopol 934p nanoemulsion	gels	Extend drug action time (sustained-release)	*in vitro*
Saroha et al.	2017	Timolol	chitosan NPs	drops	Extend drug action time (sustained-release)	*in vitro*
Maulvi et al.	2019	Timolol	Gold NPs (GNP)	contact lenses	Increased drug concentration	animal
Nagai et al.	2017	Timolol	magnesium hydroxide nanoparticles (nMH)	drops	Enhance corneal penetration ability	animal
M. Kita et al.	2021	Timolol	nMH	drops	Enhance corneal penetration ability	animal
Cuggino et al.	2021	Timolol	N-isopropylacrylamide (NIPA) and acrylic acid (AAc) nanogels	gels	Extend drug action time (sustained-release)	animal
Andreadis et al.	2022	Timolol	polyvinyl alcohol (PVA) and poloxamer 407 nanofiber	films	Extend drug action time (sustained-release)	animal
De et al.	2003	brimonidine	polyacrylic acid NPs	drops	Extend drug action time (sustained-release)	*in vitro*
Singh et al.	2010	brimonidine	sodium alginate NPs	drops	Extend drug action time (sustained-release)	animal
Ibrahim et al.	2013&2015	brimonidine	PCL, PLA, PLGA, sodium alginate NPs	drops	Extend drug action time (sustained-release)	animal
Schnichels et al.	2021	brimonidine	lipid-DNA NPs	drops	Extend drug action time (sustained-release)	animal
Maiti, Prabhu	2010&2011	brimonidine	cholesterol NPs	drops	Extend drug action time (sustained-release)	animal
Bhagav et al.	2011	brimonidine	acrylic resin NPs	drops	Extend drug action time (sustained-release), Reduce adverse reactions	animal
Khopade et al.	2022	brimonidine	resins NPs	drops	Reduce adverse reactions	animal
Barwal et al.	2019	brimonidine	chitosan NPs	drops	Extend drug action time (sustained-release)	clinical
El-Salamouni et al.	2018	brimonidine	liposomes	drops	Extend drug action time (sustained-release)	animal
Soni et al.	2021	brimonidine	cationic liposomes	drops	Enhance corneal penetration ability	*in vitro*
Sharma et al.	2020&2021	brimonidine	PLGA/PCL NPs	gels	Enhance corneal penetration ability, Extend drug action time (sustained-release)	animal
Park et al.	2015	brimonidine	PLGA/PEGs NPs	drops	Enhanced mucosal affinity and increased bioavailability	animal
Xu et al.	2022	brimonidine	silica NPs	contact lenses	Avoiding sudden drug release	animal
Sun et al.	2017	brimonidine	double-layered hydroxide NPs	contact lenses	Extend drug action time (sustained-release)	animal
Gandara-Loe et al.	2020	brimonidine	MOF NPs	films	Extend drug action time (sustained-release)	*in vitro*
Lancina et al.	2017	brimonidine	polyethyleneimine/PEG nanofiber	films	Extend drug action time (sustained-release) and increased bioavailability	animal
Natarajan et al.	2012&2014	Latanoprost	phospholipids liposomes	drops	Extend drug action time (sustained-release)	clinical
Bessone et al.	2021	Latanoprost	liposomes	drops	Extend drug action time (sustained-release)	animal
J.Tau et al.	2022	Latanoprost	nanoemulsion	drops	Reduce the application of preservatives and Reduced cytotoxicity	*in vitro*
A.M.L. Rubenicia et al.	2021	Latanoprost	Hyaluronic Acid-Chitosan NPs	drops	Reduce the application of preservatives and Extend drug action time (sustained-release)	animal
Giarmoukakis et al.	2013	Latanoprost	PLA-PEG NPs	subconjunctival injection	Extend drug action time (sustained-release)	animal
Kim et al.	2022	Latanoprost	PLGA NPs	Iontophoretic	increased bioavailability	animal
Kashiwagi et al.	2013	Latanoprost	chitosan and alginic acid Nano coating	films	Extend drug action time (sustained-release), Reduce adverse reactions	animal
Dang et al.	2022	Latanoprost	liposomes	contact lenses	Extend drug action time (sustained-release)	*in vitro*
Schnichels et al.	2020	travoprost	DNA NPs	drops	Enhance corneal penetration ability	animal
Ismail et al.	2020	travoprost	nanoemulsion	drops	Extend drug action time (sustained-release)	animal
Shukr et al.	2022	travoprost	mannitol NPs	ocular insert & gels	Extend drug action time (sustained-release)	animal
Talaeihas et al.	2020	brinzolamide	nanoemulsion	gels	Improve bioavailability	animal
Cegielska et al.	2022	brinzolamide	nanofiber	films	Enhanced mucosal affinity & Extend drug action time (sustained-release)	*in vitro*
Song et al.	2020	brinzolamide	PS-PLGA NPs	drops	Enhance corneal penetration ability	animal
Gupta et al.	2022	brinzolamide	niosomals	gels	Extend drug action time (sustained-release)	animal
Dubey et al.	2020	brinzolamide	chitosan-pectin NPs	drops	Enhance corneal penetration ability, Extend drug action time (sustained-release)	animal
Tuomela et al.	2014	brinzolamide	nanocrystals	drops	Reduced cytotoxicity	animal
Ammar et al.	2009, 2010&2022	dorzolamide	nanoemulsion	drops	Extend drug action time (sustained-release) and increased bioavailability	animal
Jóhannesson et al.	2014	dorzolamide	γ-cyclodextrin NPs	drops	increased bioavailability	clinical
Katiyar et al.	2014	dorzolamide	chitosan NPs	gels	Extend drug action time (sustained-release) and increased bioavailability	*in vitro*
Shinde et al.	2013	dorzolamide	chitosan NPs	drops	Extend drug action time (sustained-release) and increased bioavailability	*in vitro*
Warsi et al.	2014	dorzolamide	PLGA NPs	drops	Enhance corneal penetration ability	animal
Park et al.	2017	dorzolamide	PLGA-PEG Nanofiber particles	drops	Enhanced mucosal affinity & Extend drug action time (sustained-release)	animal
Mittal et al.	2019	dorzolamide	Leucaena leucocephala galactomannan NPs	drops	Enhance corneal penetration ability, Extend drug action time (sustained-release)	animal
Shahab's et al.	2022	dorzolamide	chitosan-modified polycaprolactone	drops	Enhance corneal penetration ability, Extend drug action time (sustained-release)	animal
Kouchak et al.	2018	dorzolamide	lipid carriers	drops	Enhance corneal penetration ability	clinical
Mietzner et al.	2020	fasudil	PLGA NPs	intravitreal injection	Extend drug action time (sustained-release) and increased bioavailability	*in vitro*
Bigdeli & Shrivastava et al.	2018&2023	timolol/brimonidine	Liposomal	drops	Enhance corneal penetration ability, Extend drug action time (sustained-release)	animal
Elissavet Taka et al.	2020	timolol/brimonidine	self-assembling peptide	gels	Enhance corneal penetration ability, Extend drug action time (sustained-release)	*in vitro*
Hu Yang et al.	2012	timolol/brimonidine	PLGA NPs	gels	Extend drug action time (sustained-release) and increased bioavailability	animal
Chen et al.	2022	brinzolamide/latanoprost	Nano-lipoidal	drops	Enhance corneal penetration ability, Extend drug action time (sustained-release)	clinical

## 2 Cholinergic agonists

The most commonly used cholinergic agonist in the treatment of elevated intraocular pressure is pilocarpine. Pilocarpine induces ciliary muscle contraction, which helps relax the trabecular meshwork and flatten the iris root, thereby expanding the anterior chamber angle. However, the duration of action for pilocarpine eye drops is only 4–8 h, leading to the need for frequent administration and resulting in poor patient compliance. In 1989, Diebold et al. conducted an experiment where they mixed pilocarpine with polybutyl-2-cyanoacrylate (PBCA) nanoparticles, sized at 300 nm, for a period of 72 h. This experiment resulted in a new long-acting nanoparticle formulation of pilocarpine [18]. This formulation significantly prolonged the duration of pupil constriction and reduced intraocular pressure. In a separate study, Kao et al. loaded pilocarpine into pre-prepared chitosan carbopol nanoparticles with a size of approximately 300 nm [19]. In vitro release experiments demonstrated that this nanomedicine delivery system had the longest drug release time compared to pilocarpine eye drops, pilocarpine gel eye ointment, and pilocarpine liposomes, addressing the issue of frequent drug administration. Lin et al. used chitosan polyacrylic acid nanoparticles with a size of approximately 100 nm to load pilocarpine [20]. Animal experiments showed that this drug delivery system extended the pupil contraction time from 150 min with regular pilocarpine eye drops to 315 min, significantly prolonging the topical presence of pilocarpine in the eye. Elsa et al. designed a pH (acid)-responsive pilocarpine nanoparticle drug delivery system [21]. The system consisted of polymer nanomicelles with sizes ranging from 100 to 300 nm. In 2020, Nguyen et al. and their research team invented hollow polylactic acid (PLA) nanoparticles loaded with pilocarpine, with adjustable shell thickness [22]. When injected into the anterior chamber of rabbits, the pilocarpine release time from this nano system reached up to 56 days.

The cornea, the outermost transparent tissue of the eye, has relatively low permeability, which affects the bioavailability of eye drops. One common approach to enhance corneal permeability is to use nanotechnology to prepare nanoparticles as carriers. These nanoparticles can increase the drug's permeability and absorption. Luo et al. who were part of Nguyen's team, developed a nanomedicine delivery system to improve the corneal penetration ability of pilocarpine [23]. They utilized surface-modified chitosan and ZM241385 with hollow cerium dioxide nanoparticles as carriers for loading pilocarpine (hCe-CS/ZM). The surface-modified chitosan enhanced the penetration of the nanosystem through the cornea, while ZM241385 facilitated the targeting of the nanomedicine delivery system to the desired site by binding to adenosine A2A receptors on the ciliary body. In 2022, Nguyen et al. adjusted the level of chitosan amination (concentration of amino groups on chitosan) in the pilocarpine-loaded hCe-CS nanodelivery system to regulate its corneal penetration ability [24]. Higher amination levels increased the permeability through corneal cells by 43 times. The utilization of nanomedicine delivery systems significantly enhances the topical presence and corneal penetration ability of pilocarpine, and even allows for targeted and controlled drug release.

## 3 β-Adrenergic antagonist

Timolol, a topical ocular β-adrenergic antagonist, is widely used in the treatment of glaucoma. However, studies on rabbits have revealed that the bioavailability of timolol maleate eye drops, the preferred ocular formulation, is only 1.22 % [25]. This limited bioavailability can be attributed to the low lipophilicity and poor corneal penetration ability of the formulation. To address these challenges, researchers have investigated different strategies.

Agnihotri et al. developed chitosan nanoparticles loaded with timolol in the size range of 100–200 nm. In vitro studies confirmed that these nanoparticles exhibited sustained release of the drug for a duration of 24 h [26]. Attama et al. improved the corneal permeability and release duration of timolol maleate by using surface-modified solid lipid nanoparticles as carriers [27]. Hyun et al. developed a silicone hydrogel contact lens embedded with timolol-loaded nanoparticles, enabling controlled release of the drug for up to one month at the optimal ratio [28]. Furthermore, they also developed a nanocarrier system placed in the fornix of the eyelid, achieving a maximum release time of three months [29].

Gallarate et al. conducted a study where they prepared a timolol maleate nanoemulsion using the oil-in-water (O/W) method. In vitro device tests were conducted to evaluate the effectiveness of the nanoemulsion. The results showed that the nanoemulsion not only reduced corneal irritation compared to conventional aqueous timolol but also improved corneal permeability [30]. Saroha et al. enhanced the drug release properties of timolol by encapsulating it within chitosan nanoparticles [31]. Maulvi et al. incorporated gold nanoparticles (GNPs) into contact lenses and immersed them in a timolol solution [32]. The study findings showed that the addition of GNPs did not impact the refractive performance of the lenses. However, it did significantly enhance the absorption of timolol during soaking. Additionally, the researchers introduced graphene oxide (GO) into contact lenses and immersed them in a timolol solution. This approach effectively reduced the burst release caused by direct soaking and improved the transparency and swelling characteristics of the lenses. Nagai et al. also investigated the enhancement of corneal penetration of water-soluble timolol by co-administering it with magnesium hydroxide nanoparticles (nMH) in dogs, confirming its effectiveness [33,34]. Cuggino et al. successfully loaded timolol maleate into a nanogel made with N-isopropylacrylamide and acrylic acid by utilizing ion interaction [35]. This method effectively reduced intraocular pressure in rabbit eyes for 48 h. Vijaya Rani et al. a nano in situ gel emulsion that sustained the release of timolol maleate for over 24 h [36]. Andreadis et al. utilized electrospinning to create a nanofiber membrane using polyvinyl alcohol (PVA) and poloxamer 407 as an ocular drug delivery system for timolol maleate [37]. The membrane exhibited non-irritating properties and showed a shorter onset time and longer duration of ocular pressure reduction.

Similar to pilocarpine, the limited corneal penetration and poor bioavailability of timolol maleate can be improved by enhancing its lipophilicity. Research has shown that enhancing the surface lipophilicity of the nanocarrier system allows timolol to enter the anterior chamber more easily, reduces drug loss on the ocular surface, and prolongs the drug's action time. Another strategy involves fixing the nanocarrier system onto ocular contact materials such as fornix inserts, contact lenses, and thin films to extend the drug's contact time. This can significantly increase the drug loading capacity without affecting the physical characteristics of these ocular contact materials.

## 4 α-Adrenergic agonists

Apraclonidine and brimonidine are frequently utilized α-adrenergic agonists for ocular applications. Nevertheless, the extended use of apraclonidine is restricted due to its high allergy rate and rapid tolerance [38]. Brimonidine can constrict the ciliary vessels, thereby reducing the production of aqueous humor, and enhance the outflow of aqueous humor through the uveoscleral pathway [39]. Additionally, various studies have demonstrated the neuroprotective effects of brimonidine, making it widely employed in clinical practice [[40], [41], [42]].

De et al. developed polyacrylic acid nanoparticles for the encapsulation of brimonidine. In their *in vitro* cell experiments, it was observed that the medication was gradually released over a few hours and accumulated on or between the cell surfaces [43]. Singh achieved sustained release of brimonidine for 8 h in rabbit eyes using sodium alginate nanoparticles as carriers [44]. Ibrahim prepared brimonidine-loaded nanoparticles employing PCL, PLA, PLGA, sodium alginate, and chitosan. These nanoparticles exhibited continuous and sustained drug release without any sudden spikes, effectively reducing intraocular pressure [45,46]. Maiti and Prabhu utilized nanovesicles for brimonidine delivery, achieving sustained release for 7.5 h in vivo [47,48]. Bhagav et al. found that acrylic resin nanoparticles loaded with brimonidine exhibited enhanced ocular hypotensive effects and longer duration of action compared to brimonidine eye drops. This could potentially decrease the occurrence of adverse reactions like follicular conjunctivitis, foreign body sensation, and tearing [49]. Animal experiments demonstrated that a single application of this drug delivery system yielded a comparable effect to three applications of conventional brimonidine formulations. This has the potential to reduce follicular conjunctivitis and other adverse reactions. Park et al. achieved an improvement in the adhesion of PLGA nanoparticles, which were loaded with brimonidine, to the mucosa by surface modification with a layer of PEG. Consequently, the bioavailability of brimonidine increased significantly by several folds [50]. Sun et al. successfully encapsulated brimonidine within double-layered hydroxide nanoparticles and dispersed these drug-loaded nanoparticles in a thermosensitive gel [51]. The gel was then applied to the inner side of a corneal contact lens, allowing for the continuous release of the drug for a duration of over 7 days in experimental rabbits. Lancina et al. utilized a combination of polyethyleneimine modified with PEG (polyethylene glycol) and brimonidine, which were then electrospun into nanofiber films [52]. The resulting films demonstrated prolonged reduction in intraocular pressure, surpassing the effectiveness of brimonidine eye drops. Barwal et al. incorporated chitosan nanoparticles loaded with brimonidine onto ex vivo trabecular meshwork tissue [53]. And trabecular meshwork exhibited improved relaxation compared to simple brimonidine. The *in vitro* release profile indicated a release time of up to 100 h. El-Salamouni et al. employed nanoliposomes with a size of approximately 150 nm to encapsulate brimonidine. This drug delivery system remained stable for a period of 3 months when stored at 4 °C [54]. In vivo experiments showed that it exhibited the most potent and long-lasting effect in reducing intraocular pressure. Schnichels et al. developed lipid-DNA nanoparticles loaded with brimonidine, which greatly improved corneal affinity and effectiveness [55].

Soni et al. conducted a study where they designed and optimized cationic liposomes containing brimonidine. The liposomes showed a significant increase in corneal permeability and remained stable for a period of over 3 months [56]. In a separate study, Gandara-Loe et al. developed a polyurethane film that incorporated metal-organic framework (MOF) nanoparticles. When this film was immersed in a brimonidine solution, it facilitated sustained release of the drug for up to 14 days [57]. The film had the potential to be used as a skirt for corneal contact lenses. Sharma et al. developed temperature-sensitive hydrogel-encapsulated brimonidine nanoparticles using PLGA/PCL and a surface layer of vitamin E-tocopheryl polyethylene glycol 1000 succinate (TPGS) [58,59]. Upon application to the ocular surface, the nanoparticle solution underwent transformation into an in-situ hydrogel within 20 s. This resulted in sustained release of the drug for over 24 h and a 3.5-fold increase in corneal permeability. Khopade et al. ground resins (Amberlite® IRP-69) to a size of approximately 190 nm. They then mixed the ground resins with a brimonidine solution for 30 min, resulting in the formation of a nano resin-drug complex [60]. Another study by Xu et al. utilized silica nanoparticles to encapsulate brimonidine before incorporating them into contact lenses [61]. This approach was chosen to avoid any changes in the contact lens's swelling rate, light transmittance, and oxygen permeability, and to prevent sudden drug release.

Brimonidine eye drops have low bioavailability, and using them frequently can cause local allergic reactions. However, the application of nanotechnology offers significant benefits in terms of reducing the need for frequent dosing and achieving sustained drug release.

## 5 Prostaglandin analogs

Prostaglandin analogs are a relatively new category of medications used to treat glaucoma. They work by increasing uveoscleral outflow rate, and at least one of them also increases conventional aqueous humor outflow facility as well. Due to their strong effectiveness, these medications are usually taken once a day and are considered the first choice for treatment in clinical practice [62]. However, recent studies have shown that long-term use of prostaglandin analogs can have adverse effects, such as conjunctival hyperemia, corneal damage, decreased central corneal thickness, and loss of periorbital fat [63].

### 5.1 Latanoprost

4.2 Liposomes have emerged as an established nanoscale medication delivery system. Natarajan et al. successfully developed a liposomal formulation using phospholipids that resulted in sustained release of latanoprost for up to 90 days after subconjunctival injection in monkey eyes. Notably, no inflammation was observed during this period [64]. Subsequently, the same research group conducted a clinical study involving six patients with high intraocular pressure or primary angle-closure glaucoma. The study demonstrated that this nanocarrier system also achieved a three-month reduction in intraocular pressure in humans, with favorable safety outcomes [65]. Giarmoukakis et al. employed PLA-PEG nanoparticles for the delivery of latanoprost acid to the anterior chamber, resulting in a consistent release of the drug [66]. Kashiwagi et al. designed a thin film coated with a nanolayer of chitosan and alginic acid on a PVA substrate, which demonstrated sustained release of latanoprost into the anterior chamber for a duration of six days without inducing conjunctival hyperemia [67]. Bessone et al. formulated latanoprost into liposomes, which exhibited sustained drug release and enhanced intraocular pressure-lowering effects in both *in vitro* and in vivo experiments [68]. Additionally, cell studies compared a latanoprost nanoemulsion with conventional formulations containing the preservative BAK, and the nanoemulsion demonstrated lower cytotoxicity [69]. Researchers have also investigated the use of hyaluronic acid-chitosan nano systems for delivering latanoprost in order to decrease the need for BAK, prolong the duration of its effects, and improve the effectiveness of reducing intraocular pressure [70]. Kim et al. conducted a study where they first attached PLGA nanoparticles loaded with latanoprost to the ocular surface. They then used an electrode to quickly attract the nanoparticles into the cornea. This single administration approach resulted in a prolonged duration of action lasting for seven days and demonstrated 23 times higher drug efficacy compared to commercial formulations [71]. Dang et al. conducted a study where they investigated the impact of loading latanoprost into polyethylene glycol-modified solid lipid nanoparticles and incorporating them into a corneal contact lens. The researchers found that this process did not affect important properties of the contact lens, such as transparency and swelling. However, it did significantly increase the capacity of the lens to hold the drug, allowing for a high-dose release of latanoprost over a 96-h period [72].

### 5.2 Travoprost

Schnichels et al. developed DNA nanoparticles that incorporated nucleic acid aptamers to bind with travoprost. This resulted in enhanced ocular adhesion and a drug release rate that was twice as fast as that of eye drops at different time intervals [73]. Ismail et al. focused on preparing a travoprost nanoemulsion using triglycerides, which effectively improved ocular drug absorption and prolonged the duration of intraocular pressure reduction [74]. Shukr et al. utilized elastic nanovesicles based on mannitol to transport travoprost and formulated them into ocular inserts for sustained drug release [75].

In contrast to the aforementioned intraocular pressure-lowering medications, prostaglandin analogs have longer carbon chains in their chemical structure, making them highly lipophilic (with good corneal penetration ability) but poorly water-soluble. Therefore, efforts should be directed towards enhancing their intraocular distribution to improve efficacy and minimize drug-related side effects when developing nanoscale formulations. Currently, limited research has been conducted on nano formulations of other prostaglandin analogs such as travoprost, tafluprost, and unoprostone. However, exploring these analogs in nanocarrier-based drug delivery holds potential for breakthroughs.

## 6 Carbonic anhydrase inhibitors

Brinzolamide and dorzolamide are commonly used ocular carbonic anhydrase inhibitors. These medications work by inhibiting carbonic anhydrase in the ciliary epithelium, which reduces the rate of production of aqueous humor and lowers intraocular pressure. However, long-term use of brinzolamide (administered as brinzolamide eye drops) often leads to adverse reactions such as altered taste sensation, ocular discomfort (including burning sensation, stinging, itching, and transient blurred vision), foreign body sensation, and ocular congestion [76]. In addition to addressing these adverse reactions, nanotechnology holds promise in improving the solubility of brinzolamide (currently in a suspension form), reducing the frequency of administration required for brinzolamide, and adjusting the pH of dorzolamide hydrochloride eye drops.

### 6.1 Brinzolamide

Talaei has developed a nanoemulsion formulation containing brinzolamide that transforms into an in-situ water gel upon contact with ocular tissues like the cornea and conjunctiva [77]. This transformation increases the drug's residence time on the ocular surface, potentially improving its effectiveness. Animal experiments have also shown that this nano formulation is highly safe and well-tolerated by the eyes. Cegielska et al. have employed electrospinning technology to fabricate nanofiber films containing brinzolamide [78]. These nanofiber films can be directly applied onto the cornea for drug delivery, providing a more accurate administration approach in comparison to conventional eye drops. Song et al. developed core-shell structured Brz-PS-PLGA nanoparticles with a size of approximately 500 nm using coaxial electro-spraying [79]. The incorporation of phosphatidylserine (PS) in the shell greatly improves the penetration ability of this drug delivery system through the cornea. Dubey et al. have developed chitosan-pectin nanocapsules loaded with brinzolamide [80]. This system effectively prolongs the drug's presence in the cornea, enhances its permeability, and enables sustained release, ultimately improving its bioavailability. Gupta et al. developed an in-situ gel nano-vesicle formulation that effectively reduces intraocular pressure for up to 24 h, thus decreasing the need for frequent administration of brinzolamide [81]. Tuomela et al. synthesized brinzolamide nanocrystals, which exhibit low ocular toxicity and enhanced water solubility of brinzolamide, as demonstrated by *in vitro* and in vivo studies [82]. In addition, some researchers have modified brinzolamide chemically by attaching hydrophobic groups to its amino moiety through an acylation reaction. Nanoparticles prepared using the precipitation method have shown significantly improved corneal permeability [83].

### 6.2 Dorzolamide

A study conducted by Ammar et al. involved the design and preparation of 17 different nanoemulsion eye drops containing dorzolamide [84]. These nanoemulsion formulations consisted of various oils, surfactants, and co-surfactants. Through experiments on rabbit eyes, the researchers observed that these nanoemulsion eye drops demonstrated a faster onset of action, longer drug release time, and no ocular irritation when compared to conventional dorzolamide eye drops. Following this, the same research team developed a nanoemulsion formulation that could form an in-situ gel on the corneal surface, resulting in improved bioavailability [85]. In 2021, they employed response surface methodology to optimize the design of a cationic nanoemulsion loaded with dorzolamide, which also exhibited similar delivery advantages [86]. Jóhannesson and Gudmundsdottir conducted a study to assess the safety of γ-cyclodextrin nanoparticles eye drops in rabbit eyes. They later tested dorzolamide-loaded γ-cyclodextrin nanoparticles eye drops on human eyes. The results showed that these nanoparticles effectively reduced intraocular pressure, similar to commercial eye drops, but with improved safety [87,88]. Katiyar developed a chitosan nanocrystal in situ gel measuring 164 nm in size. The gel demonstrated good corneal retention time and sustained drug release [89]. Shinde prepared chitosan nanoparticles loaded with dorzolamide, which exhibited sustained drug release and significantly improved bioavailability [90]. Warsi et al. developed PLGA nanoparticles loaded with dorzolamide, showing no ocular irritation and improved corneal permeability and drug concentration in the aqueous humor [91]. Park et al. utilized electrospinning to create nanofibers of PLGA-PEG containing dorzolamide, which were then ground into particles [92]. This system demonstrated significantly enhanced mucosal adhesion, resulting in prolonged ocular residence time and more than a twofold increase in the duration of intraocular pressure reduction compared to commercial dorzolamide eye drops. Mittal et al. developed biodegradable polymer nanoparticles loaded with dorzolamide using Leucaena leucocephala seeds as a raw material. These nanoparticles exhibited high corneal permeability and sustained drug release [93]. In another study, Shahab et al. encapsulated dorzolamide in chitosan-modified polycaprolactone nanoparticles, achieving a 12-h sustained release [94]. The mucoadhesion strength increased by 3.7 times, and corneal permeability was significantly improved. Additionally, they encapsulated dorzolamide in solid lipid nanoparticles, resulting in sustained release for 2–10 days and a 2.87-fold increase in corneal permeability. Kouchak et al. conducted a study involving 20 patients with primary open-angle glaucoma (POAG) or ocular hypertension (OHT) [95]. They observed that the intraocular pressure of patients using dorzolamide-loaded nanostructured lipid carriers was lower than those using commercial dorzolamide eye drops on the 14th and 28th days. The discussion and analysis suggested that the improved corneal penetration of the system was attributed to the phospholipid bilayer, smaller particle size, and positive zeta potential of the nanostructured lipid carriers.

Similar to other ocular hypotensive drugs, nanotechnology-based formulations aim to reduce the frequency of administration and improve bioavailability by enhancing corneal retention and penetration of the drug. In the case of brinzolamide, the application of novel nano formulations can improve its water solubility, transforming the existing suspension into a stable solution dosage form. This ensures consistency during drug administration. For dorzolamide, which has an acidic commercial formulation (buffer pH 5.6), the use of nano formulations can alleviate discomfort and ocular side effects associated with excessively low pH levels.

## 7 ROCK inhibitors

The novel drugs ROCK inhibitors ripasudil and netarsudil were approved for the treatment of glaucoma in Japan in 2014 and in the United States in 2018, respectively. These drugs may lower intraocular pressure through two mechanisms: affecting the endothelial cells of the Schlemm's canal to increase intercellular gaps, thus improving trabecular aqueous humor outflow facility, or inducing relaxation of the trabecular meshwork smooth muscle fibers, thereby also improving trabecular aqueous humor outflow facility. However, the application of ROCK inhibitors topically leads to a high incidence of hyperemia and low intraocular bioavailability. Research has demonstrated that it is possible to enhance the bioavailability and efficacy of ROCK kinase inhibitors by using nanoparticles. For instance, PLGA nanoparticles that encapsulate the ROCK inhibitor fasudil have shown significant improvements in the drug's stability and bioavailability, ultimately enhancing its therapeutic effect [96].

Due to the novelty of these drugs, there is currently limited research available on their nanoscale formulations. However, once extensive clinical studies have been conducted to determine their effectiveness and potential side effects, nanotechnology can be employed to tailor their formulations for more appropriate ocular applications.

## 8 Other intraocular pressure lowering drugs

Fixed combination formulations show promise for treating glaucoma. However, traditional combinations have not yielded optimal therapeutic results due to poor ocular bioavailability of the drugs. In addition to the mentioned classes of intraocular pressure lowering drugs, researchers have also investigated the use of nanotechnology to enhance combination formulations, which has shown promising outcomes. One example is the development of liposomal nanocarriers for timolol maleate/brimonidine fixed combination formulations. These nanocarriers demonstrate improved release patterns, permeation, and significant reduction in intraocular pressure compared to simple aqueous solutions [97,98].

Elissavet Taka et al. developed an in-situ self-assembling peptide hydrogel that delivers timolol and brimonidine to the eye simultaneously [99]. This innovative system achieved rapid and complete release of both drugs within 8 h and increased corneal permeability by 2–5 times. Hu Yang designed a novel hybrid platform using polyamidoamine dendrimer hydrogel and PLGA nanoparticles to co-deliver timolol and brimonidine [100]. Compared to conventional formulations, this platform maintained significantly higher concentrations of brimonidine in the aqueous humor, cornea, and conjunctiva for up to 7 days. Importantly, this formulation did not cause ocular inflammation or discomfort. The hybrid platform demonstrated the ability to enhance drug bioavailability and sustain effective reduction of intraocular pressure over an extended period of time.

Furthermore, micelle polymerization was utilized to produce polyalkylcyanoacrylate nanoparticles loaded with pilocarpine and timolol, presenting a novel drug delivery approach [101]. Nano-lipoidal carriers loaded with brinzolamide and latanoprost demonstrated effective reduction of intraocular pressure and remarkable transcorneal permeation [102]. Alginate and chitosan-based nanosheets loaded with latanoprost/timolol exhibited a slow and sustained reduction of intraocular pressure [103]. Wenpei Fan et al. designed hollow mesoporous organosilica nanocapsules for the efficient co-delivery of hydrophobic JS-K and hydrophilic l-Arginine [104]. This innovative device achieved nitric oxide release through activating endothelial nitric oxide synthase in the trabecular meshwork and Schlemm's canal microenvironment, resulting in a significant reduction of intraocular pressure in various glaucoma mouse models. Regarding fixed-dose combinations, although nanotechnology can enhance drug release patterns and permeability, further research and improvement are still necessary.

## 9 Discussion

The concentration of locally administered eye drops that reach the eye is extremely low. Research has shown that only 1–4% of the dose reaches the anterior chamber of the eye, and less than 0.1 % reaches the retina. The penetration of local eye drops into the eye primarily occurs through corneal and non-corneal routes. The non-corneal route includes the conjunctiva, sclera, choroid, and retina, and drug absorption through the tissue vessels in this route is believed to undergo a similar process of ‘first-pass elimination.’ Therefore, the corneal penetration is considered the primary route for drugs to enter the eye. Specifically, locally administered drugs that penetrate the cornea must first dissolve in the tear film or mucus on the ocular surface, then traverse the lipophilic corneal epithelium, enter the corneal stroma, and finally penetrate the corneal endothelium to reach the eye. It can be inferred that highly water-soluble or highly lipophilic drug molecules cannot effectively enter the eye. Therefore, drugs need to have a certain degree of water solubility to achieve a sufficient concentration in the tear film, as well as a certain degree of lipid solubility to facilitate rapid penetration of the corneal epithelium. The utilization of nanotechnology in ocular drug delivery systems can significantly enhance the corneal residence time and corneal penetration of drugs by providing appropriate water/lipid solubility and surface charge of nanoparticles, thereby improving the drug's bioavailability. Improved bioavailability not only increases the dose of the drug that reaches the eye, thereby enhancing its effectiveness, but also reduces the dose of the drug that does not reach the eye, minimizing ocular surface side effects caused by the drug. However, certain drugs, such as the β-adrenergic receptor antagonist Timolol, still face challenges in current nanodelivery systems due to their low lipid solubility and poor corneal permeability.

The application of nanomaterials in the sustained release of glaucoma drugs is currently in the exploratory stage, requiring further research to verify and optimize these new technologies and methods, particularly in clinical practice. Safety and biocompatibility are crucial considerations. Published literature on nano drug delivery systems often emphasizes their favorable safety and biocompatibility due to publication bias. Common toxicities associated with nanomaterials include immunotoxicity, genetic toxicity, and epigenetic toxicity [105]. Immunotoxicity, extensively discussed, primarily refers to the adverse effects of nanomaterials on the immune system [106]. Current research suggests that nanomaterials mainly impact the innate immunity of the immune system, involving cells such as dendritic cells, monocytes, neutrophils, and NK cells. However, the specific effects remain uncertain. Some nanomaterials have the potential to inhibit immune responses, weakening the body's self-protection ability, while others may stimulate the immune system, leading to unnecessary inflammation and rejection reactions. Fortunately, the eyeball is often considered an immune-privileged organ, offering promising prospects for applying nanotechnology in ophthalmology. Despite the uncertainties surrounding the impact of nanomaterials on the immune system, the unique characteristics of the eye suggest a bright future for the application of nanotechnology in ophthalmic contexts.

The majority of nano eye drops designed to lower intraocular pressure are still in the animal experimental stage. Only a few, such as liposomes and cyclodextrin, have progressed to human trials, where their effectiveness and safety have been confirmed [64,65,88,95]. The safety of liposomes is inherently evident, as exosomes represent a natural type of liposome. Additionally, cyclodextrin is a recognized safe food and drug excipient. This suggests that selecting appropriate substrates and utilizing nanotechnology in the development of eye drops offers a more secure approach. However, the safety of other nanomaterials, such as those based on metals, carbon, and silicon, requires further validation. The efficacy and potential risks associated with these materials need to be thoroughly examined before considering them secure for application in eye drops.

### 9.1 Prospect

Nanotechnology offers new possibilities for drug delivery, including the use of nanoparticles in corneal contact lens-related drug delivery systems. By using nanoparticles, it is possible to effectively address changes in refractive parameters, swelling coefficient, and oxygen permeability that are typically associated with direct drug doping. Moreover, this approach also allows for an increase in the drug-loading capacity of the system. For example, Kim et al. demonstrated the successful delivery of nanoparticles into the eye through a technique called ‘corneal electrophoresis' [71].

There is still significant potential for advancement in nanotechnology. Currently, scientists are not only satisfied with encapsulating drugs using lipid membranes, but they are also investigating the expression of specific proteins on the membrane surface. They are utilizing protein-containing lipid membranes to achieve precise targeted drug delivery, which further enhances the “unit drug efficacy".

The use of nano delivery systems for lowering intraocular pressure has demonstrated significant potential in the treatment of eye diseases like glaucoma. These systems can enhance drug permeability and residence time in eye tissues, leading to improved treatment effectiveness and reduced frequency of drug administration. Future research in this field could concentrate on various aspects including targeted therapy, persistent and controlled release of drugs, ensuring biocompatibility and safety, developing multifunctional nano delivery systems, and exploring clinical applications and translation of these systems.

Targeted therapy aims to enhance treatment effectiveness while minimizing damage to healthy tissues by precisely delivering drugs to the specific tissues or cells in the eye that need treatment. This is achieved through the utilization of surface functionalization technology, where specific ligands, antibodies, or drugs are incorporated onto the surface of nanoparticles. These functionalized nanoparticles can then selectively bind to receptors or biomarkers on the target cells' surface, facilitating targeted drug delivery.

Persistent and controlled release: In order to ensure a slow and prolonged release of drugs in eye tissues, it is common practice to encapsulate them in sustained-release carriers using controllable release technology. A promising approach is the development of nano delivery systems that can respond to specific environmental conditions. For instance, nanoparticles can be designed to be sensitive to physiological conditions that are specific to eye diseases, such as pH, temperature, enzyme activity, etc. This enables the achievement of controlled drug release under specific conditions.

Further research and resolution are necessary to address the safety and biocompatibility concerns surrounding nanodelivery systems. It is essential to select nanocarriers made of materials with high biocompatibility, ensuring they do not cause inflammatory reactions or other adverse effects in the eye environment. The design of nanocarriers should take into account factors such as stability, biodegradability, and their impact on eye tissue. Additionally, the drugs carried by nanomaterials must undergo rigorous safety assessments. Understanding the distribution, metabolism, and clearance pathways of nanodelivery systems in the body is crucial for evaluating their safety and predicting potential toxic reactions. This knowledge can help optimize the design of delivery systems.

The multifunctionality of nano delivery systems for lowering intraocular pressure refers to their ability to perform multiple functions, such as anti-inflammatory, antioxidant, anti-fibrosis, and promoting cell regeneration. These nano delivery systems can release multiple drugs simultaneously or sequentially, and they have multiple therapeutic mechanisms to improve treatment efficacy and cover various therapeutic targets. By designing and adjusting a multifunctional nano delivery system according to the specific condition of the patient, personalized treatment can be achieved, leading to improved treatment effectiveness. It is important to design and optimize these systems considering the characteristics, release mechanisms, and drug interactions of different drugs.

Clinical application and translation: Nanodelivery systems present new opportunities for developing improved, convenient, and personalized treatment strategies. However, further research and validation are necessary to enable their widespread use in clinical practice. During the preclinical research phase, it is essential to conduct clinical trials to assess the safety, efficacy, and tolerability of nanocarrier systems. These steps require adherence to regulations, comprehensive market analysis, and well-defined commercialization plans to ensure successful market entry and widespread acceptance of the nanodelivery system.

The comprehensive application of these methods and technologies can increase the residence time of drugs in eye tissues, reduce the frequency of drug use, and improve treatment effectiveness and patient experience. Persistent optimization will be crucial in the nano delivery system of intraocular pressure lowering drugs to meet long-term treatment needs. The design and optimization of the nano delivery system for lowering intraocular pressure drugs should be based on the characteristics, release mechanisms, and drug interactions of different drugs. This requires interdisciplinary research in fields such as pharmacology, biomedical engineering, and nanotechnology. In the future, multifunctional nano delivery systems have broad application prospects in the treatment of eye diseases, offering patients more effective and personalized treatment plans.

## 10 Conclusion

Research on nano delivery systems for intraocular pressure lowering drugs offers new opportunities for the development of more effective, convenient, and personalized therapy strategies in ophthalmology. These nano delivery systems have the potential to improve drug efficacy, prolong the duration of drug action, reduce the frequency of drug administration and improve patient compliance. However, it is important to note that research on nano delivery systems is still in the developmental phase, and further validation and optimization of various technologies and methods are required. Additionally, ensuring the safety and biocompatibility of these nano delivery systems is a crucial concern that needs to be addressed.

## Funding

This work was supported by the 10.13039/501100001809National Natural Science Foundation of China [grant numbers 81970801]; Hunan Engineering Research Center for Glaucoma with Artificial Intelligence in Diagnosis and Application of New Materials [grant numbers 2023TP2225]; the Natural Science Foundation of Hunan Province, China [grant numbers 2023JJ40004, 2023JJ40003, 2023JJ70014]; Changsha Municipal Natural Science Foundation [grant numbers kq2208495] and the Science and Technology Foundation of Aier Eye Hospital Group, China [grant numbers AR2206D4, AR2206D2, AR2206D5 and Aier Glaucoma Institute]; Hunan Province “Little Lotus” science and technology talent special fund [grant numbers 2023 TJ-X24].

## Method

### Literature search statement

Independent literature search was conducted by two authors in PubMed, Google Scholar, Web of Science. The search strategy included the following terms: “cholinergic agonists,” “β-adrenergic antagonist,” “α-adrenergic agonists,” “prostaglandin analogs,” “carbonic anhydrase inhibitors,” “ROCK inhibitors,” “pilocarpine,” “timolol,” “brimonidine,”“latanoprost,” “travoprost,” “bimatoprost,” “brinzolamide,” “dorzolamide,” “intraocular pressure lowering,” “glaucoma,” and “nano.”

## Data availability statement

No data was used for the research described in the article.

## CRediT authorship contribution statement

**Xiaoyu Zhou:** Writing – review & editing, Writing – original draft, Funding acquisition, Conceptualization. **Dengming Zhou:** Writing – original draft, Methodology, Conceptualization. **Xinyue Zhang:** Investigation, Funding acquisition. **Yang Zhao:** Writing – review & editing. **Li Liao:** Writing – review & editing. **Ping Wu:** Investigation. **Baihua Chen:** Writing – review & editing. **Xuanchu Duan:** Writing – review & editing, Supervision, Funding acquisition.

## Declaration of competing interest

The authors declare that they have no known competing financial interests or personal relationships that could have appeared to influence the work reported in this paper.
